# *ICOS* DNA methylation regulates melanoma cell-intrinsic *ICOS* expression, is associated with melanoma differentiation, prognosis, and predicts response to immune checkpoint blockade

**DOI:** 10.1186/s40364-023-00508-2

**Published:** 2023-06-01

**Authors:** Damian J. Ralser, Emmanuelle Herr, Luka de Vos, Zsófi Kulcsár, Romina Zarbl, Niklas Klümper, Gerrit H. Gielen, Alexander Philippe Maas, Friederike Hoffmann, Jörn Dietrich, Pia Kuster, Alexander Mustea, Nicole Glodde, Glen Kristiansen, Sebastian Strieth, Jennifer Landsberg, Dimo Dietrich

**Affiliations:** 1 Department of Gynaecology and Gynaecological Oncology, University Medical Center Bonn (UKB), Bonn, Germany; 2 Institute of Experimental Oncology, University Medical Center Bonn (UKB), Bonn, Germany; 3 Department of Otorhinolaryngology, University Medical Center Bonn (UKB), Venusberg-Campus 1, 53127 Bonn, Germany; 4 Department of Dermatology and Allergology, University Medical Center Bonn (UKB), Bonn, Germany; 5 Department of Urology, University Medical Center Bonn (UKB), Bonn, Germany; 6 Institute of Neuropathology, University Medical Center Bonn (UKB), Bonn, Germany; 7 Institute of Pathology, University Medical Center Bonn (UKB), Bonn, Germany

**Keywords:** ICOS, *ICOS* DNA methylation, Epigenetics, Immune checkpoint blockade, Melanoma, Predictive biomarker

## Abstract

**Background:**

Inducible T cell costimulator ICOS is an emerging target in immuno-oncology. The aim of this study was to investigate the epigenetic regulation of *ICOS* in melanoma by DNA methylation.

**Methods:**

We comprehensively investigate *ICOS* DNA methylation of specific CpG sites and expression pattern within the melanoma microenvironment with regard to immune correlates, differentiation, clinical outcomes, and immune checkpoint blockade (ICB) response.

**Results:**

Our study revealed a sequence-contextual CpG methylation pattern consistent with an epigenetically regulated gene. We found a cell type-specific methylation pattern and locus-specific correlations and associations of CpG methylation with *ICOS* mRNA expression, immune infiltration, melanoma differentiation, prognosis, and response to ICB. High *ICOS* mRNA expression was identified as a surrogate for enriched immune cell infiltration and was associated with favorable overall survival (OS) in non-ICB-treated patients and predicted response and a prolonged progression-free survival (PFS) following ICB therapy initiation. *ICOS* hypomethylation, however, significantly correlated with poor OS in non-ICB patients but predicted higher response and prolonged PFS and OS in ICB-treated patients. Moreover, we observed cytoplasmic and sporadically nuclear tumor cell-intrinsic ICOS protein expression. Tumor cell-intrinsic *ICOS* protein and mRNA expression was inducible by pharmacological demethylation with decitabine.

**Conclusion:**

Our study identified *ICOS* DNA methylation and mRNA expression as promising prognostic and predictive biomarkers for immunotherapy in melanoma and points towards a hitherto undescribed role of ICOS in tumor cells.

**Supplementary Information:**

The online version contains supplementary material available at 10.1186/s40364-023-00508-2.

## Background

Melanoma represents the most aggressive form of cutaneous cancer with increasing incidence and mortality rates worldwide. While for many years there were only limited therapeutic modalities with a severely limited prognosis, the therapeutic armamentarium has substantially expanded due to a deeper understanding of melanoma biology and particularly the introduction of therapeutics targeting the immune system. Immune checkpoint blockade (ICB), in particular blockade of the immune checkpoints cytotoxic T-lymphocyte-associated protein 4 (CTLA-4), programmed cell death protein 1 (PD-1), and lymphocyte-activation gene 3 (LAG-3), has revolutionized the treatment of advanced melanoma with significantly improved therapy response and long-term survival (reviewed in: [1]). However, despite these recent improvements, patients display highly variable treatment responses encompassing complete remission, secondary resistance, and primary resistance. Furthermore, immune-mediated side effects occur in a large proportion of patients during ICB treatment with considerable morbidity and mortality [1]. Considering the numerous further innovative immunotherapies currently being in preclinical and clinical development, including inhibitors targeting other immune checkpoints, cytokines, modulators of metabolic pathways, cell therapies, therapeutic vaccines or combinations of the aforementioned agents, biomarker-driven therapy management is an overriding goal in modern oncology.

One emerging immunotherapeutic target is the inducible T cell costimulator (ICOS, cluster of differentiation 278 [CD278]) [2, 3]. ICOS is expressed by different T lymphocyte subpopulations, including CD8^+^ activated cytotoxic T lymphocytes (CTLs), CD4^+^ T helper (Th) cells, and CD4^+^, FoxP3^+^ regulatory T cells (Tregs) and has been implicated in various immunological processes such as T cell differentiation and T cell response [3]. The ligand of ICOS, ICOSL (CD275), is primarily expressed by antigen-presenting cells (APCs), including B cells, macrophages and dendritic cells. Further, ICOSL is expressed on the surface of endothelial cells, lung epithelial cells, fibroblasts, mesenchymal stem cells, and tumour cells [4]. In the oncological context, ICOS/ICOSL interaction leads to both, antitumor response via activation of CTLs and Ths, and protumor immunosuppressive response mediated by Tregs [2, 3]. In melanoma, ICOS is the most expressed costimulatory receptor by tumor-infiltrating lymphocytes (TILs) with ICOS positivity found on regulatory, CD4^+^, and CD8^+^ T cells [5]. Of note, the prognostic value of ICOS expression was dependent on the T cell subtype expressing ICOS. High ICOS expression by Tregs was associated with poor prognosis [6], whereas high expression on non-Treg TILs was linked to a better outcome [7, 8]. Regarding expression by tumor cells, research has shown, that ICOSL is expressed by melanoma cells promoting the expansion of Tregs via ICOS/ICOSL signaling [4]. Thus, ICOSL expression by melanoma tumor cells could favor immune escape by driving Treg expansion and survival.

With regard to response to immunotherapy, data suggests that ICOS could enhance the efficacy of CTLA-4-directed therapy. In patients with bladder cancer, breast cancer, non-small cell lung cancer (NSCLC), and melanoma treated with anti-CTLA-4 immunotherapy, increased levels of ICOS^+^CD4^+^ T cells in tumor tissue and peripheral blood were detected [9–12]. Presence of ICOS^+^CD4^+^ T cells was associated with a good therapy response [13]. Of note, another study demonstrated enhanced PI3K signalling in these ICOS^+^CD4^+^ T cells from anti-CTLA-4-treated patients [14]. ICOS and ICOSL deficient mice displayed impaired antitumor responses after treatment with CTLA-4-directed ICB [15]. Considering the contrasting, pro- and antitumoral dual function of ICOS/ICOSL, ICOS represents both, a promising target for immunotherapy and an interesting biomarker in terms of immunotherapy response and prognosis.

Agonistic and antagonistic monoclonal antibodies (mAbs) targeting ICOS are currently under clinical and preclinical investigation [3]. Agonistically acting mAbs are expected to enhance the antitumor CD4^+^ and CD8^+^ T cell immune response, whereas antagonistically acting antibodies are expected to suppress Treg function and thus reverse the immune escape. Research has shown that the effect of CTLA-4- or PD-1/PD-L1-directed ICB is enhanced by adding agonistic ICOS mAb. INDUCE-1 (ClinicalTrials.gov Identifier: NCT02723955) was the first clinical trial evaluating these synergistic effects of targeting both, PD-1 (pembrolizumab) and ICOS (GSK3359609, feladilimab, GlaxoSmithKline) in patients. This basket trial showed promising antitumor activity and a tolerable toxicity profile. In the subgroup of patients with heavily pre-treated advanced melanoma, feladilimab demonstrated clinical activity applied as both, monotherapy and in combination with pembrolizumab. Of note, the majority of patients were already pre-treated with anti PD-(L)1 ICB. This benefit was further validated in the ICONIC trial (NCT02904226) for a second ICOS agonistic mAb (JTX-2011, Jounce Therapeutics). A large number of further studies are currently investigating the clinical efficacy of agonistic anti-ICOS mAbs in various malignancies, including melanoma, either applied as monotherapy (NCT03447314, NCT02904226) or in combination with ICB and chemotherapy (NCT03693612, NCT03447314, NCT02723955, NCT03739710, NCT02904226, NCT04128696, NCT04428333). For antagonistic ICOS mAbs, only limited antitumoral activity has been demonstrated so far with several clinical trials being underway (NCT02520791, NCT03829501).

However, the decision regarding treatment with agonistic or antagonistic ICOS mAbs should depend on the patient-specific tumor microenvironment. In the presence of ICOS^+^ Tregs, the application of antagonistic mAbs would be reasonable in terms to reverse immune escape, whereas in the presence of ICOS^+^CD8^+^ T cells, the antitumor effect should be enhanced by administration of agonistic mAbs. In this context, identification of robust, mechanistically driven biomarkers is of the highest priority.

In recent years, epigenetic signatures have increasingly become the focus of interest in immuno-oncological biomarker research. Epigenetics, in particular DNA methylation, plays a major role in the development of resistance to ICB, T cell exhaustion, and T cell rejuvenation [16, 17]. Methylation has been shown to regulate expression of CTLA-4, PD-1, and PD-L1 in various malignancies [18–20]. Recently, we provided evidence for *CTLA4* methylation to serve as a predictive biomarker for anti-PD-1 and anti-CTLA-4 ICB in patients with metastatic melanoma and metastatic clear cell renal cell carcinoma [21–23].

Little is known regarding the epigenetic regulation of *ICOS* in melanoma. The crucial role of ICOS for anti-tumor response elicited by CTLA-4-directed ICB renders ICOS as a potential biomarker to predict CTLA-4-directed ICB response and further for the stratification of patients who may benefit from immunotherapeutic ICOS-directed treatment. Here, we aimed to comprehensively investigate *ICOS* gene methylation at single CpG resolution and expression pattern with regard to transcriptional activity, patients’ survival and response to immunotherapy, and tumor microenvironment.

## Methods

### Patients

#### TCGA cohort

Comprehensive methylation, expression, immunogenomic, and clinicopathological data from *N* = 470 patients from The Cancer Genome Atlas Research Network (TCGA, http://cancergenome.nih.gov/) melanoma cohort were analyzed retrospectively. A detailed cohort characterization has been published earlier by the TCGA Research Network [24]. Patients had signed informed consent prior to registration in accordance with the declaration of Helsinki principles.

#### UHB ICB case/control set

Formalin-fixed and paraffin-embedded (FFPE) tumor tissues from *N* = 48 patients with advanced melanoma who were treated with anti-PD-1 ICB at the University Hospital Bonn (UHB) were retrospectively included as previously described [25]. *N* = 19 experienced early disease progression following initiation of ICB, whereas *N* = 29 displayed durable remission. Patients’ baseline characteristics are shown in Supplemental Table S1. The study was approved by the local institutional review board.

#### UHB ICB cohort

We included a cohort comprised of FFPE tumor tissues from *N* = 123 patients with advanced melanoma who were treated with anti-PD-1 ICB at the UHB between 2012 and 2022. A limitation of this cohort represents the depletion of *N* = 19 patients with progressive disease and *N* = 29 patients with durable remission who were included into the UHB ICB case/control set. The study was approved by the local institutional review board.

#### Liu et al*.* ICB cohort

mRNA expression data from *N* = 121 melanoma patients treated with anti-PD-1 monotherapy or anti-PD-1 plus anti-CTLA-4 combination therapy were included in the study. Retrospective data was obtained from Liu et al. [26].

#### Isolated cell populations (Tirosh et al. and Hannon et al*.*)

Further, methylation data on peripheral isolated leukocytes, comprising monocytes, B cells, CD4^+^ T cells, CD8^+^ T cells, and granulocytes, of *N* = 28 healthy patients were provided by Hannon et al*.* [27] (Gene Expression Omnibus (GEO) accession number: GSE103541; National Center for Biotechnology Information (NCBI), Bethesda, MD). Single-cell RNA-sequencing data comprising *N* = 4,645 single cells including malignant, immune, and stromal cells isolated from *N* = 19 melanoma tissue was provided by Tirosh et al. [28].

#### Tonsillar tissue

Fresh frozen tonsillar tissues were obtained from the BioBank Bonn of the UHB.

### Clinical endpoints

Overall survival (OS) was defined as time from date of biospecimen collection/accession to date of last follow-up or death (TCGA cohort) [24]. Patients with events after 60 months were censored at 60 months because after five years of observation deaths might be unrelated to melanoma. ICB therapy response (progressive disease, stable disease, partial response, complete response) was measured according to RECIST 1.1 criteria [29]. Progression-free survival (PFS) in the ICB therapy context (Liu et al*.* ICB cohort and UHB ICB case/control set) was defined as the time from ICB initiation until date of last follow-up, objective tumor progression or death, respectively.

### Cell Lines

Data on methylation and mRNA expression from *N* = 33 melanoma cell lines were obtained from The Genomics of Drug Sensitivity in Cancer (GDSC) database [30] (https://www.cancerrxgene.org/).

The human melanoma cell line A375 (RRID:CVCL_0132) was obtained from American Type Culture Collection (ATCC, Manassas, VA, USA). Authentification of this cell line was performed by the ‘Leibniz-Institut DSMZ-Deutsche Sammlung von Mikroorganismen und Zellkulturen GmbH’ (Braunschweig, Germany). Mycoplasma contamination testing were performed on a regular basis. A375 cells were cultured in complete RPMI 1640 medium (cat. no. 21875059, Thermo Fisher Scientific, Waltham, MA, USA) supplemented with 10% [v/v] fetal bovine serum (FBS, heat inactivated, cat. no. FBS. S 0615HI, Bio&SELL GmbH, Nuremburg, Germany), 1X MEM (Minimum Essential Medium) Non-Essential Amino Acids Solution (100X stock, cat. no. 11140035, Thermo Fisher Scientific), 1 mM HEPES (1 M stock, cat. no. 15630056, Thermo Fisher Scientific), 1 mM 2-mercaptoethanol (cat. no. 21985023, Thermo Fisher Scientific), 100 U/ml penicillin and streptomycin (10,000 U/ml stock, cat. no. 15140122, Thermo Fisher Scientific), and 1 mM sodium pyruvate (100 mM stock, cat. no. 11360070, Thermo Fisher Scientific). A375 cells were treated with demethylating 10 μM 5-aza-2-deoxycytidine (decitabine, 5-aza-dC; cat. no. ab120842, Abcam, Cambridge, UK) for 168 h. Untreated A375 cells were used as control. The growth medium was changed every 24 h.

### Methylation analysis

DNA methylation data from *N* = 470 patients generated by the TCGA Research Network using Infinium HumanMethylation450 BeadChip (Illumina, Inc., San Diego, CA, USA) were downloaded from the Genomic Data Commons (GDC) Data Portal (https://portal.gdc.cancer.gov) [24]. Methylation data from peripheral isolated leukocytes (*N* = 28) and melanoma cell lines (*N* = 33) were collected applying the HumanMethylation450 BeadChip and downloaded from the Gene Expression Omnibus webpage (GSE103541) and GDSC database, respectively. Methylation levels were calculated as *β*-values as described previously [31]. *β*-values were multiplied with the factor 100% in order to represent estimates of approximate percent methylation (0% to 100%). Methylation data of the UHB ICB case/control set (*N* = 48) was generated using the HumanMethylationEPIC BeadChip. *β*-values of methylation levels were calculated for seven BeadChip beads that target CpG sites within the promoter, promoter flanks, intragenic regions, and up- and downstream sequences of *ICOS*: cg18219180 (CpG 1), cg00372692 (CpG 2), cg21423458 (CpG 3), cg18561976 (CpG 4), cg15344028 (CpG 5), cg 15,247,069 (CpG 6), and cg17751550 (CpG 7).

*ICOS* methylation levels in UHB ICB cohort was determined using a quantitative methylation specific PCR (qMSP) that target the CpG sites 4 and 5. Bisulfite converted DNA from tumor tissues was prepared using the EpiTect Fast Bisulfite Kit (Qiagen, Hilden, Germany) according to the manufacturer’s instructions. The PCR buffer was formulated as previously described [32] and cycling was done for 20 min at 95 °C (polymerase activation) followed by 45 PCR cycles (62 °C / 2 s, 54 °C / 60 s, 95 °C / 15 s). The qMSP contained two methylation-unspecific primers that did not target CpG sites and two methylation-specific probes that hybridize competitively to CpG sites 4 and 5 (forward primer: CTTCCTTTCCAACAAATAAAAAACA (0.4 μM), reverse primer: GTAAGTAGAAGAGAAAGAAATATTAGA (0.4 μM), probe_methylated_: 6-FAM-AACTTTAAACACTAAA**CGCG**AAAA-BHQ-1 (0.2 μM), probe_unmethylated_: HEX-TCAACTTTAAACACTAAA**CACA**AAAAC-BHQ-1 (0.2 μM)). Cycle threshold (CT) values were used to calculate Quantitative Methylation Scores (QMS) were computed as follows: QMS = 100%/(1 + 2^(CT_methylated_ − CT_unmethylated_)) [21].

### mRNA expression analysis

Transcript abundance represented by RSEM (RNA-Seq by Expectation Maximization) normalized counts (n.c.) of *N* = 468 patients from the TCGA cohort were generated using the Illumina HiSeq 2000 RNA Sequencing approach [24]. mRNA expression data (Robust Multi-array Average [RMA] levels) from *N* = 33 melanoma cell lines (Human Genome U219 Array, Affymetrix, Santa Clara, CA, USA) were obtained from the GDSC database [30]; https://www.cancerrxgene.org/). Additional RNA sequencing data from the Liu et al. cohort (*N* = 121) were provided by Liu et al. [26].

We used quantitative reverse transcription PCR (qRT-PCR) to assess *ICOS* mRNA levels in A375 melanoma cell lines treated with decitabine compared to untreated cell lines and tonsillar tissues. We used five housekeeping genes (*ACTB*, *SDHA*, *HPRT1*, *ALAS1*, *GAPDH*) for quantification of total mRNA. RNA extraction was performed using the NucleoSpin RNA Mini kit (Macherey Nagel, Düren, Germany, cat. no. 740955) according to the manufacturer’s instructions. We used the HiScript II Q RT SuperMix for qPCR (Vazyme, Nanjing, China, cat. no. R222) for cDNA synthesis. Quantification by qRT-PCR was conducted using PCR buffer conditions as described above. Oligonucleotides and cycling conditions: *ICOS* – forward primer: ACTTGGACCATTCTCATGCC (0.4 μM), reverse primer: CCTATGGGTAACCAGAACTTCA (0.4 μM), probe: Atto 647N-ATTTATGAAGTATTCATCCAGTGTGC-BHQ-2 (0.2 μM), PCR cycling: 20 min / 95 °C and 45 PCR x [54 °C / 60 s, 95 °C / 15 s]; *ACTB* – forward primer: ATGTGGCCGAGGACTTTGATT (0.2 μM), reverse primer: AGTGGGGTGGCTTTTAGGATG (0.2 μM), probe: HEX-GAAATRMGTKGTTACAGGAAGTCCCT-BHQ-1 (wobble bases: R – A/G, M – A/C, K – G/T) (0.16 μM), 20 min / 95 °C and 25 PCR x [58 °C / 60 s, 95 °C / 15 s]; *SDHA* – forward primer: TCGCTCTTGGACCTGGT (0.2 μM), reverse primer: TGGAGGGACTTTATCTCCAG (0.2 μM), probe: 6-FAM-ATCGAAGAGTCATGCAGGCC-BHQ-1 (0.16 μM), 20 min / 95 °C and 25 PCR x [62 °C / 60 s, 95 °C / 15 s]; *HPRT1* – forward primer: TGACACTGGCAAAACAATGCA (0.2 μM), reverse primer: GGTCCTTTTCACCAGCAAGCT (0.2 μM), probe: 6-FAM-TGCTTTCCTTGGTCAGGCAGTAT-BHQ-1 (0.16 μM), 20 min / 95 °C and 25 PCR x [62 °C / 60 s, 95 °C / 15 s]; *ALAS1* – forward primer: TAATGACTACCTAGGAATGAGTCG (0.2 μM), reverse primer: CCATGTTGTTTCAAAGTGTCCA (0.2 μM), probe: 6-FAM-TAACTGCCCCACACACCCGT-BHQ-1 (0.16 μM), 20 min / 95 °C and 25 PCR x [62 °C / 60 s, 95 °C / 15 s]; *GAPDH* – forward primer: TGCACCACCAACTGCTTAGC (0.2 μM), reverse primer: GGCATGGACTGTGGTCATGAG (0.2 μM), probe: 6-FAM-CTGGCCAAGGTCATCCATGACAACT-BHQ-1 (0.16 μM), 20 min / 95 °C and 25 PCR x [58 °C / 60 s, 95 °C / 15 s]. We computed mean CT values of all five reference genes (CT_reference genes_) and ΔCT values were calculated (ΔCT_sample_ = CT_reference genes_ – CT_*ICOS*_).

### Immune signatures

Data on lymphocyte distribution (0–3; 0 = no lymphocytes within the tissue, 1 = lymphocytes present in < 25% of the tissue, 2 = lymphocytes present in 25 to 50% of the tissue, 3 = lymphocytes present in > 50% of tissue), lymphocyte density (0–3; 0 = absent, 1 = mild, 2 = moderate, 3 = severe), and lymphocyte score (0–6, score defined as the sum of the lymphocyte distribution and lymphocyte density scores) were obtained from TCGA [24]. Lymphocyte score in the UHB ICB case/control set was assessed accordingly. *CD8A*, *CD8B*, *CD4*, *CD19*, *CD20*, and *CD14* RNA-Seq data were used as quantitative measure for CD8^+^ T cell, CD4^+^ T cell, B cell, and monocytes, respectively.

### Immunohistochemistry

Immunohistochemical staining (IHC) for ICOS protein expression was performed in the UHB ICB case/control set. In brief, 4 μm thick paraffin sections were cut from the original FFPE tissue block and subsequently stained using the Dako Omnis system (Dako / Agilent Technologies) applying a rabbit monoclonal anti-ICOS antibody (clone SP98, 1:25 dilution, RRID:AB_10710236, cat. no. ab105227, abcam, UK). We performed antigen retrieval with target retrieval solution at pH 6 for 10 min at 117 °C in a steam pressure cooker. Slide-incubation with the primary antibody was performed overnight at 4 °C. Signal detection was performed with an Alkaline Phosphatase Red Detection Kit (Dako / Agilent Technologies, cat. no. K5005). The slides were finally counterstained with hematoxylin and bluing reagent, dehydrated, and mounted. Tonsillar tissue was used as positive control. Tumoral ICOS protein expression was evaluated applying the H-score (negative (0), weak (1), moderate (2), and strong (3) ICOS expression (H-score: 0–300)). ICOS^+^ immune cells were assessed as percentage fraction from all cells (ICOS^+^ lymphocyte score).

### Flow cytometry

A375 melanoma cell line pellets (untreated and 5-aza-dC treated) were washed with flow cytometry buffer (1X Dulbecco’s Phosphate Buffered Saline [cat. no. 14190094, Thermo Fisher Scientific], 4% [v/v] FBS, 2 mM ethylenediaminetetraacetic acid [EDTA]). Single cell suspensions were stained with the following fluorochrome-conjugated antibodies: anti-human ICOS (clone SP98, 1:100 in flow cytometry buffer) and LIVE/DEAD™ Fixable Near-IR Dead Cell Stain Kit (cat. no. L10119, Thermo Fisher Scientific, 1:1,000 in flow cytometry buffer). We used rabbit IgG isotype control (RRID:AB_2532938, cat. no. 02–6102, Thermo Fisher Scientific) for reference staining. Flow cytometry data were acquired with a FACSCanto™ Flow Cytometer (Becton, Dickinson and Company, NJ, USA) and analyzed with FlowJo software (version 10.8.0, Becton, Dickinson and Company).

### Statistics

Statistical analyses were performed using IBM SPSS® Statistics Version 27.0.0.0 (IBM Corp., Armonk, NY, USA). Non-parametric Spearman’s *ρ* correlation coefficients were calculated. Group comparisons were made using parametric two-sided Student’s *t*-test, paired *t*-test, nonparametric Mann–Whitney *U* (two groups) or Kruskal–Wallis (> 2 groups) test. Survival analyses of median dichotomized variables were performed using the log-rank test and visualized via Kaplan–Meier plots. Continuous variables were used for Cox proportional HR analyses with specified 95% confidence intervals (95%CI). *P*-values < 0.05 were considered statistically significant.

## Result

### *ICOS* expression pattern in melanoma

**Fig. 1:**
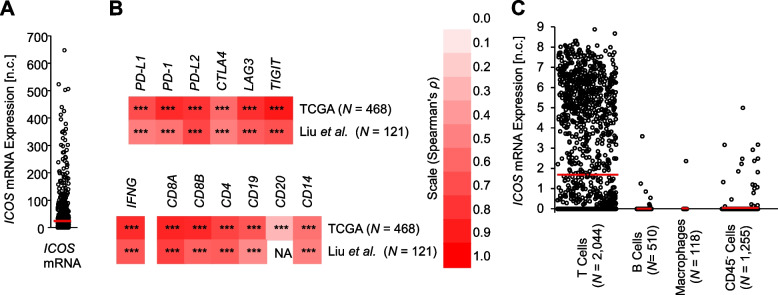
*ICOS* mRNA expression within melanomas. **A** *ICOS* mRNA expression levels in *N* = 468 melanomas obtained from the TCGA cohort. **B** Correlations (Spearman’s *ρ*) of *ICOS* mRNA expression with mRNA levels of the immune checkpoint genes *PD-L1*, *PD-1*, *PD-L2*, *CTLA4*, *LAG3*, and *TIGIT*, with interferon γ mRNA expression, and with mRNA signatures of immune cell infiltrates (CD8^+^ T cells: *CD8A* and *CD8B*, CD4^+^ T cells: *CD4*, B cells: *CD19* and *CD20*, monocytes: *CD14*) in the TCGA and Liu et al*.* melanoma cohorts [24, 26]. **C** Single-cell RNA sequencing data obtained from Tirosh et al*.* [28] showing *ICOS* mRNA expression levels in T cells, B cells, macrophages, and CD45^−^ cells*.* ***P < 0.001, NA: not available

*ICOS* mRNA showed variable expression levels ranging from 0 to 647.89 n.c. (mean 59.13 n.c., median 24.83 n.c., 95%CI 51.02–67.23 n.c., *N* = 468) (Fig. 1A) in the TCGA cohort. *ICOS* mRNA expression in those whole tumor samples strongly correlated with mRNA expression of other therapeutically relevant immune checkpoint genes, i.e. *PD-L1* (*ρ* = 0.704, *P* < 0.001), *PD-1* (*ρ* = 0.843, *P* < 0.001), *PD-L2* (*ρ* = 0.800, *P* < 0.001), *CTLA4* (*ρ* = 0.586, *P* < 0.001), *LAG3* (*ρ* = 0.817, *P* < 0.001), and *TIGIT* (*ρ* = 0.906, *P* < 0.001) (Fig. 1B). We validated these significant correlations in a second independent cohort comprised of *N* = 121 patients published by Liu et al*.* [26] (Liu et al*.* ICB cohort; *PD-L1*: *ρ* = 0.511, *P* < 0.001; *PD-1*: *ρ* = 0.552, *P* < 0.001; *PD-L2*: *ρ* = 0.732, *P* < 0.001; *CTLA4*: *ρ* = 0.504, *P* < 0.001; *LAG3*: *ρ* = 0.638, *P* < 0.001; *TIGIT*: *ρ* = 0.689, *P* < 0.001; (Fig. 1B)). We found a significant positive correlation between *ICOS* mRNA and histopathologic lymphocyte score in the TCGA cohort (*ρ* = 0.473, *P* < 0.001, *N* = 328). Accordingly, *ICOS* mRNA correlated with signatures of immune infiltrates, i.e. interferon γ (*IFNG*) mRNA expression, and mRNA expression of *CD8A*, *CD8B*, *CD4*, *CD19*, *CD20*, and *CD14*, representing CD8^+^ T cell, CD4^+^ T cell, B cell, and monocytes, respectively (TCGA cohort – *IFNG*: *ρ* = 0.832, *CD8A*: *ρ* = 0.859, *CD8B*: *ρ* = 0.848, *CD4*: *ρ* = 0.740, *CD19*: *ρ* = 0.668, *CD20*: *ρ* = 0.288, *CD14*: *ρ* = 0.555, all *N* = 468 and *P* < 0.001; Liu et al*.* cohort – *IFNG*: *ρ* = 0.668, *CD8A*: *ρ* = 0.753, *CD8B*: *ρ* = 0.624, *CD4*: *ρ* = 0.642, *CD19*: *ρ* = 0.459, *CD20*: not available, *CD14*: *ρ* = 0.503, all *N* = 121 and *P* < 0.001; Fig. 1B). For a deeper understanding of *ICOS* mRNA expression by different cell types within the melanoma microenvironment, we analyzed single-cell RNA sequencing data obtained from Tirosh et al. [28]. Concordant with the well-acknowledged expression by activated T cells [33, 34] we observed highest *ICOS* mRNA expression levels in isolated T cells and only sporadic low-level expression in B cells and macrophages (Fig. 1C). Of note, we also detected *ICOS* mRNA copies in few CD45^−^ cells suggesting an *ICOS* mRNA expression by melanoma cells (Fig. 1C). In order to exclude that this signal might originate from contaminating non-melanoma cells within the CD45^−^ cell fraction, we analyzed *ICOS* mRNA expression in *N* = 33 melanoma cell lines obtained from GDSC. In melanoma cell lines, *ICOS* mRNA showed variable expression levels ranging from 0 to 2.44 RMA (mean 0.42 RMA, median 0.057 RMA, 95%CI 0.19–0.65 RMA, *N* = 33), confirming a tumor-intrinsic mRNA expression of *ICOS*. CTLA-4 protein and *CTLA4* mRNA expression by melanoma cells has been reported [22, 35]. Since both genes are co-located on chromosome 2 q33.2, are members of the CD28 immune checkpoint receptor family, are co-expressed in melanomas from the TCGA cohort, we expected co-expression of *ICOS* and *CTLA4* in melanoma cell lines as well. Despite the low number of cell lines we were able to confirm this hypothesis (*ρ* = 0.385, *P* = 0.027, *N* = 33).

**Fig. 2:**
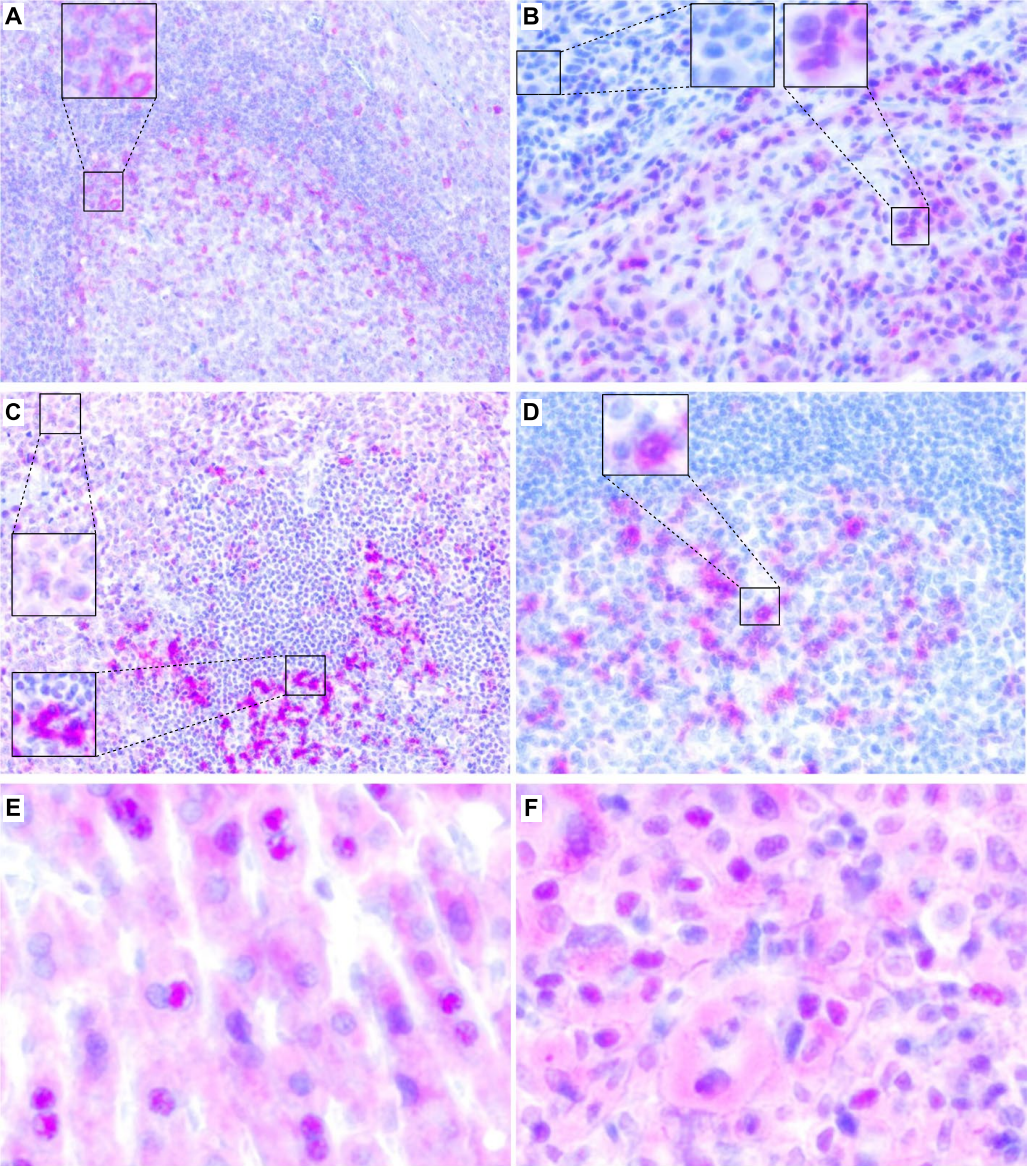
ICOS protein expression in tonsillar, melanoma, and hepatic tissue. Representative immunohistochemical staining patterns in a (**A**) tonsil, (**B**-**D**, **F**) various melanomas, and hepatocytes (**E**). **A** In tonsillar tissue, lymphatic germinal centers stain strongly positive in proximity to the mantle zone. **B** Melanoma lung metastasis with heterogeneous ICOS expression in tumor cells ranging from absent to strong. **C** Cutaneous melanoma metastasis with ICOS^+^ and ICOS^−^ TILs and moderately ICOS^+^ tumor cells. **D** Melanoma lymph node metastasis with negative lymphocytes and several strongly ICOS^+^ tumor cells. **E** Adjacent hepatocytes from a melanoma liver metastasis with moderate cytoplasmic and sporadic strong nuclear ICOS expression. **F** Melanoma with moderate cytoplasmic and sporadic strong nuclear ICOS expression

We further corroborated melanoma cell-intrinsic ICOS expression at protein level via IHC. Tonsillar tissue was used as positive control, where we observed strong positive staining of cell subsets in germinal centres in proximity to the mantle zone (Fig. 2A). In melanoma tissues from a previously described case/control set of pre-therapeutic samples from melanoma patients receiving ICB [25], we were able to confirm ICOS protein expression in subsets of melanoma cells. However, ICOS staining was rather cytoplasmic than membranous (Fig. 2B-D). Interestingly, in adjacent hepatocytes from one liver metastasis sample and melanoma cells from one brain metastasis, we also detected sporadic strong nuclear expression (Fig. 2E-F).

### Pharmacological demethylation induces ICOS expression in melanoma cells

**Fig. 3:**
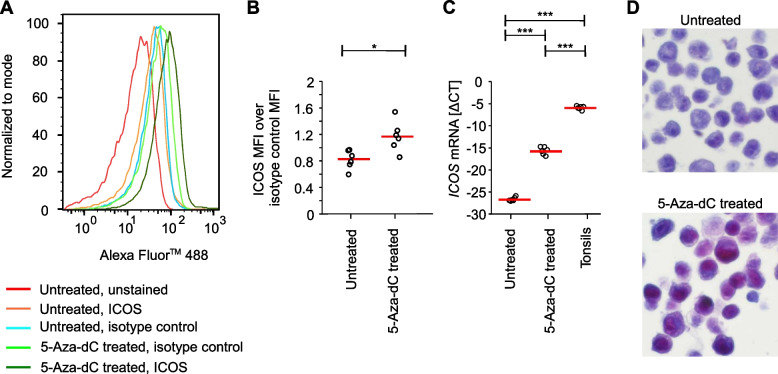
Pharmacological demethylation induces ICOS expression in melanoma cells. **A** Normalized histograms illustrating induction of ICOS expression in pharmacologically demethylated (5-aza-dC treated) compared to untreated A375 melanoma cell line (unstained, anti-ICOS, and isotype control). **B** ICOS expression (mean fluorescence intensity [MFI]) over isotype control of 5-aza-dC treated compared to untreated A375 melanoma cells (six replicates). **C** *ICOS* mRNA (ΔCT levels) expression in 5-aza-dC treated compared to untreated A375 melanoma cells (six replicates) and six individual tonsils. **D** Representative ICOS IHC of 5-aza-dC treated compared to untreated A375 melanoma cells (40 × magnification). Bars represent mean values. *P* values refer to paired *t*-tests (*P < 0.05; ***P < 0.001)

We have previously shown that the melanoma-cell intrinsic expression of the immune checkpoint receptor *TIGIT* is pharmacologically inducible by 5-aza-dC, indicating an epigenetic regulation via DNA methylation [36]. We therefore investigated the epigenetic regulation of *ICOS* via DNA methylation in more detail using the human A375 melanoma cell line. A375 melanoma cells displayed low to absent *ICOS* baseline protein and mRNA expression shown by flow cytometry, qRT-PCR, and IHC (Fig. 3). However, after pharmacological demethylation using 5-aza-dC, *ICOS* protein and mRNA expression was induced, albeit on a significantly lower level compared to tonsillar tissue used as positive control **(**Fig. 3). The effect of the 5-aza-dC treatment on ICOS protein expression as determined via flow cytometry was only moderate compared to the increase of *ICOS* mRNA levels (Fig. 3A-C), indicating a predominantly non-membranous ICOS expression pattern. Accordingly, we confirmed a mainly nuclear ICOS staining after 5-aza-dC treatment via IHC (Fig. 3D).

### *ICOS* methylation pattern in melanoma

**Fig. 4:**
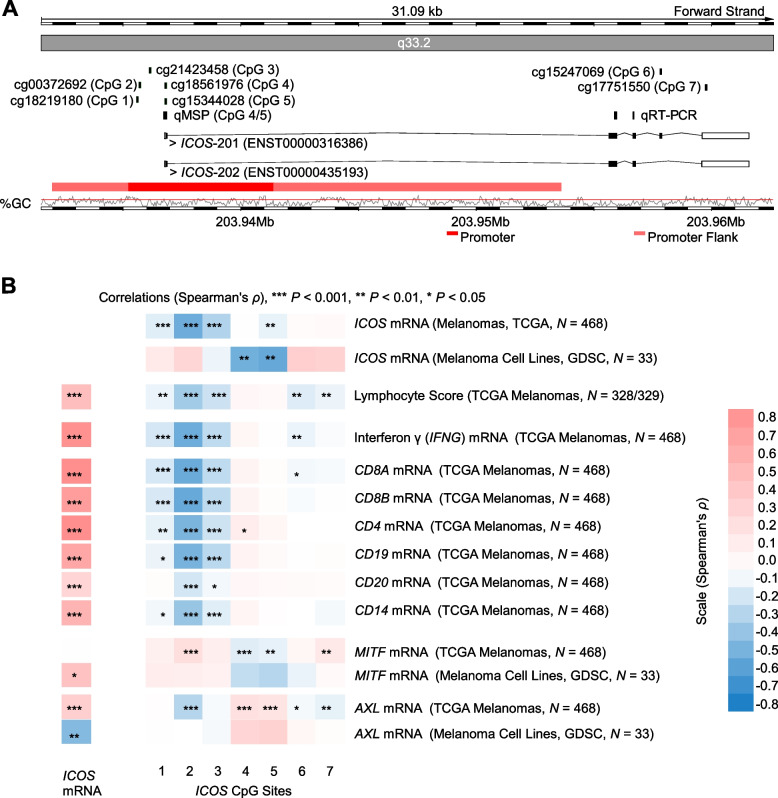
*ICOS* DNA methylation correlates with *ICOS* mRNA expression, immune cell infiltration, interferon γ, and tumoral differentiation in melanoma. **A** Genomic organization of the *ICOS* gene locus (*ICOS* transcripts, regulatory elements, guanosine/cytosine (GC)-density). Target sites of the Infinium BeadChip beads (CpG sites 1–7), qMSP (CpG site 4/5), and qRT-PCR are depicted. The illustration (modified) was exported from www.ensemble.org (Ensembl Release 109) and is based on Genome Reference Consortium Human Build 38 patch release 13 (GRCh38.p13). **B** Spearman’s correlations (Spearman’s *ρ*) of *ICOS* mRNA expression and *ICOS* DNA methylation levels obtained from the TCGA melanoma cohort and The Genomics of Drug Sensitivity in Cancer (GDSC) database (*N* = 33 melanoma cell lines; [30]; https://www.cancerrxgene.org/). Further, *ICOS* mRNA expression and *ICOS* DNA methylation status in the TCGA melanoma cohort was correlated with the lymphocyte score according to TCGA, with interferon γ (*IFNG*) mRNA, RNA-Seq signatures of immune cell infiltrates (CD8^+^ T cells: *CD8A* and *CD8B*, CD4.^+^ T cells: *CD4*, B cells: *CD19* and *CD20*, monocytes: *CD14*), and with melanoma differentiation based on *MITF* and *AXL* mRNA expression. *P < 0.05; **P < 0.01; ***P < 0.001

**Fig. 5:**
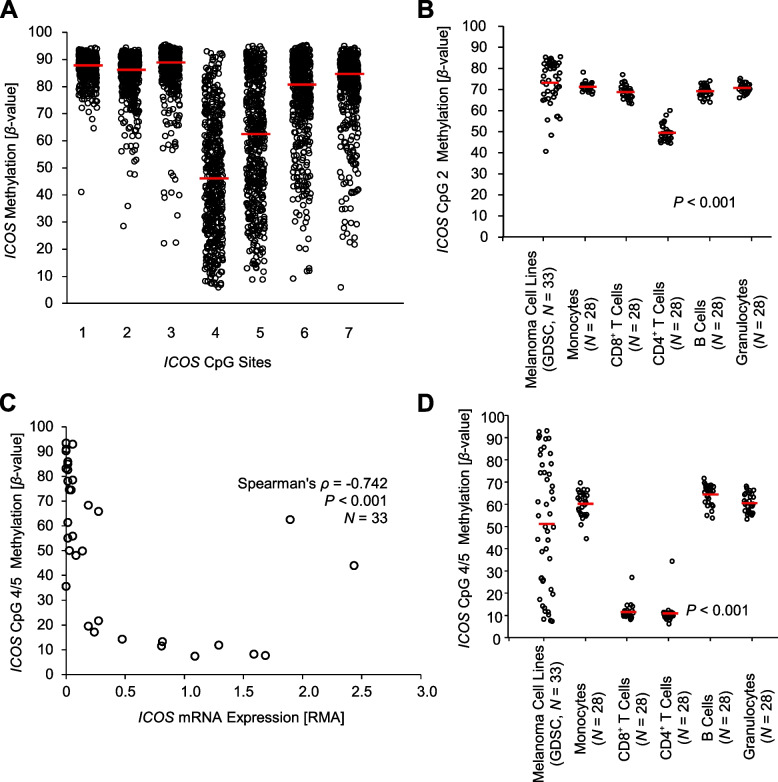
*ICOS* DNA methylation in melanoma and immune cells. **A** *ICOS* DNA methylation status [*β*-values] of seven CpG sites (CpG sites 1–7; Fig. 4A) in the melanoma TCGA cohort (*N* = 468; [24]) are shown. **B** *ICOS* DNA methylation status of CpG 2 in peripheral isolated leukocytes, comprising monocytes, B cells, CD4^+^ T cells, CD8^+^ T cells, and a granulocytes fraction (GEO accession number: GSE103541) and melanoma cell lines [30]. **C** Correlation between *ICOS* DNA methylation [*β* values] of CpG 4/5 with *ICOS* mRNA expression in melanoma cell lines. **D** *ICOS* DNA methylation status of CpG 4/5 in peripheral isolated leukocytes and melanoma cell lines. Bars represent median mRNA expression and methylation levels

Since 5-aza-dC does not lead to a locus-specific but global hypomethylation, we further investigated DNA methylation of seven specific CpG sites (CpG 1–7) with the *ICOS* gene locus. CpG sites 1–3 are located within the central promoter region upstream of the transcription start site. CpG sites 4 and 5 are found within the central promoter region overlapping with the untranslated region of the first exon, and CpG sites 6 and 7 are located within the gene body (Fig. 4A). In the TCGA cohort (*N* = 470), we found high median methylation levels (> 80%) in the upstream central promoter region (CpG sites 1–3; CpG 1: mean 87.03%, median 87.89%, 95%CI 86.58–87.47%, range 41.19–94.39%; CpG 2 mean 84.13%, median 86.43%, 95%CI 83.35–84.90%, range 28.67–94.49%; CpG site 3: mean 86.26%, median 89.11%, 95%CI 85.37–87.16%, range 22.29–95.63%; Fig. 5A). The two CpG sites 4 and 5, located within the central promoter region and the untranslated first exon, showed significantly lower mean methylation levels < 70% (CpG 4: mean 47.38%, median 46.31%, 95%CI 45.41–49.36%, range 6.09–93.14%; CpG 5 mean 60.39%, median 62.52%, 95%CI 58.34–62.43%, range 8.88–95.03%*;* Fig. 5A). For CpG sites 6 and 7, located within the *ICOS* gene body, again high median methylation levels > 80% were found [CpG 6: mean 76.06%, median 80.80%, 95%CI 74.54–77.57%, range 9.23–95.36%; CpG 7: mean 79.61%, median 84.73%, 95%CI 78.29–80.93%, range 6.04–95.08%; *N* = 470; Fig. 5A).

### *ICOS* transcriptional activity is negatively correlated with DNA methylation

To investigate to what extent the transcriptional activity of *ICOS* is regulated via DNA methylation, we correlated methylation levels at single CpG site resolution with *ICOS* mRNA expression. In the TCGA cohort (*N* = 468), CpG sites 1–3 and 5, respectively, showed a significant negative correlation of DNA methylation status with mRNA expression which was most pronounced at CpG site 2 (CpG 1: *ρ* = -0.157, *P* = 0.001; CpG 2: *ρ* = -0.536, *P* < 0.001; CpG 3: *ρ* = -0.312, *P* < 0.001; CpG 5: *ρ* = -0.132, *P* = 0.004). For the methylation of CpG sites 4, 6 and 7, respectively, no significant correlations with *ICOS* mRNA expression were observed (Fig. 4B). Since T cells are the main source of *ICOS* mRNA (Fig. 1C) and CpG 2 methylation showed the strongest negative correlation with mRNA expression levels in the heterogeneous tumor tissue, we expect lower methylation levels in T cells compared to other immune cells and melanoma cells. Accordingly, we found lower methylation levels in isolated immune cells, particularly in CD4^+^ T cells (data previously published by de Vos et al*.* [37]) compared to melanoma cells (Fig. 5B). Concordantly, we detected a strong negative correlation of CpG 2 methylation with mRNA expression of *CD8A*, *CD8B*, *CD4*, *CD19*, *CD20*, and *CD14*, surrogates for CD8^+^ T cell, CD4^+^ T cell, B cell, and monocytes, respectively (Fig. 4B). Hence, CpG 2 methylation represents a surrogate measure for infiltrating immune cells.

The high *ICOS* expression by T cells precludes the study of tumor cell-specific correlation analyses of CpG methylation and mRNA expression in heterogeneous tumors from the TCGA cohort. We therefore analyzed melanoma cell lines from GDSC. In *N* = 33 melanoma cell lines, a contrasting picture compared to whole tumor tissues from the TCGA cohort emerged: Methylation of CpG sites 1–2, and 6–7 was positively correlated with *ICOS* mRNA expression, not reaching statistical significance though, whereas methylation status of CpG sites 3–5 showed negative correlation coefficients that were statistically significant for CpG site 4 (*ρ* = -0.735, *P* < 0.001) and CpG site 5 (*ρ* = -0.750, *P* < 0.001; Fig. 4B). Since CpG sites 4 and 5 are tandem CpG sites (CACTGAA**CGCG**AGGACTG), we performed further analyses applying the mean methylation levels of both CpGs (referred to as CpG 4/5). The strong negative correlation between *ICOS* CpG 4/5 methylation and mRNA expression (*ρ* = -0.742, *P* < 0.001) is illustrated in Fig. 5C. We then compared CpG 4/5 methylation in melanoma cell lines and isolated immune cells (data previously published by de Vos et al*.* [37]) (Fig. 5D). Melanoma cell lines showed an equally distributed methylation over the whole spectrum ranging from < 10% to > 90% methylation (range 7.36–93.37%, mean 51.34%, median 52.53%, 95%CI 42.75–59.95%, *N* = 33) while isolated immune cells showed a homogeneous methylation pattern. Of note, CD8^+^ and CD4^+^ T cells showed only low-level methylation compared to other immune cells. Accordingly, CpG 4/5 methylation in a heterogeneous tumor represents in part a measure for *ICOS* mRNA expressing tumor cells. However, within the UHB ICB case/control set we did not find a significant correlation between CpG 4/5 methylation and ICOS protein expression measured by IHC (*ρ* = -0.038, *P* = 0.81).

### *ICOS* DNA methylation status is associated with melanoma differentiation

The TCGA melanoma cohort was sub-grouped into well-differentiated and poorly differentiated tumors based on expression of the differentiation drivers microphthalmia transcription factor (MIFT) and AXL, a member of the TAM family of receptor tyrosine kinases. Research has shown that AXL expression is associated with melanoma dedifferentiation, whereas MITF expression is linked to differentiated tumors [38, 39]. CpG 4/5 methylation, correlated inversely with the degree of differentiation (*MITF* mRNA: *ρ* = -0.148, *P* = 0.001) and positively with the degree of dedifferentiation (*AXL* mRNA: *ρ* = 0.180, *P* < 0.001; Fig. 4B) in the TCGA cohort. An opponent correlation pattern is also evident for CpG site 2 (*MITF* mRNA: *ρ* = 0.211, *P* < 0.001; *AXL* mRNA: *ρ* = -0.304, *P* < 0.001). Since we identified CpG 2 as a surrogate measure for immune cell infiltration, this is in-line with reports on the causal relationship between dedifferentiation and inflammation [40–43]. This was further corroborated by a positive correlation of *ICOS* mRNA with *AXL* mRNA expression (0.346, *P* < 0.001; Fig. 4B). Moreover, we found significant correlations between *ICOS* mRNA with *AXL* and *MITF* mRNA expression in the melanoma cell lines from the GDSC (*ICOS*/*AXL*: *ρ* = -0.457, *P* = 0.008; *ICOS*/*MITF*: *ρ* = 0.441, *P* = 0.010; *N* = 33).

### *ICOS* mRNA expression and methylation is associated with prognosis and response to ICB

**Fig. 6:**
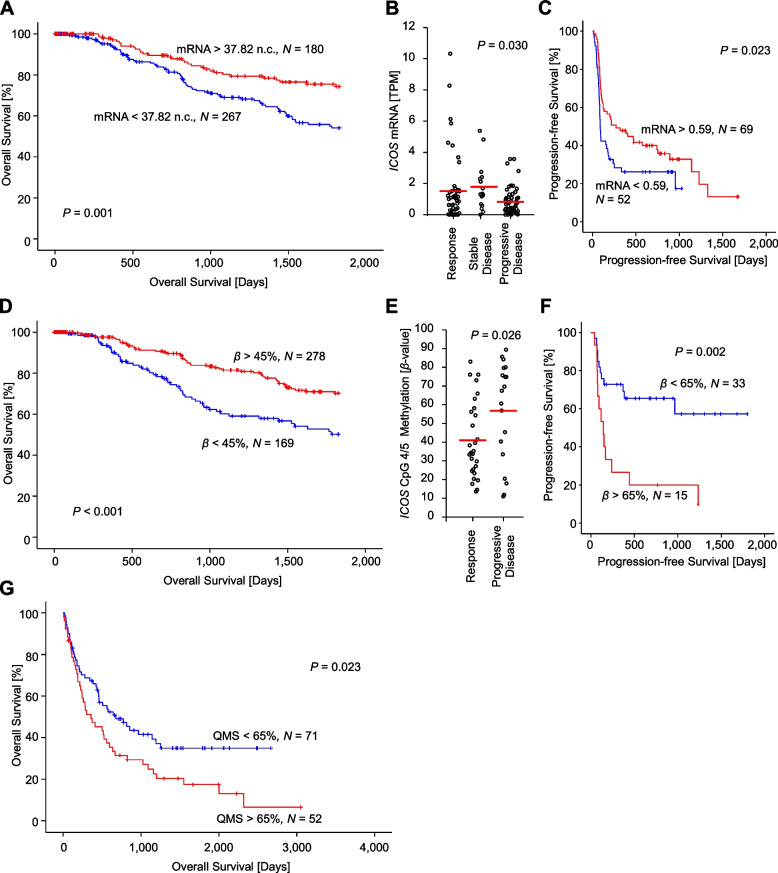
*ICOS* DNA methylation status and mRNA expression is associated with survival and predictive for ICB response in melanoma. Overall survival in the TCGA melanoma cohort stratified according to (**A**) *ICOS* DNA methylation level at CpG 4/5 and (**D**) *ICOS* mRNA expression. To preclude a prognostic bias due to ICB administration, all patients who received anti-PD-1 or anti-CTLA-4 (*N* = 23) ICB were excluded from analysis. **B** *ICOS* mRNA expression is predictive of ICB therapy response and (**C**) associated with a prolonged PFS after ICB therapy initiation (Liu et al. cohort; *N* = 121; [26]). **E** *ICOS* DNA methylation levels at CpG sites 4/5 predicts ICB treatment response and (F) is associated with prolonged progression-free survival (PFS) following ICB treatment initiation (UHB ICB case/control set; *N* = 48). **G** High *ICOS* CpG 4/5 methylation is associated with adverse overall survival in the UHB ICB cohort (*N* = 123). *P* values refer to log-rank tests, *t* test, and ANOVA, respectively. Bars represent mean values

Next, we analyzed the association between *ICOS* mRNA expression, CpG 2 methylation, and CpG 4/5 methylation with prognosis and response to ICB. We analyzed the prognostic value in the melanoma TCGA cohort. To preclude a prognostic bias due to an ICB related survival advantage, we excluded all patients who received anti-PD-1 or anti-CTLA-4 (*N* = 23) ICB. Further analyses were performed for *N* = 447 patients from the TCGA cohort. In Cox proportional hazard analysis with *ICOS* mRNA expression as a continuous variate (no cut-off to avoid overfitting), we found a weak but significant association with a prolonged OS (HR = 0.996 [95%CI 0.993–0.999], *P* = 0.006). Figure 6A visualizes this prognostic value by means of Kaplan–Meier survival analysis based on an optimized cut-off for patients’ classification. We then investigated the predictive value of *ICOS* mRNA expression in a cohort of *N* = 121 melanoma patients treated with immunotherapy published by Liu et al*.* (Liu et al. cohort; [26]). In this cohort, we confirmed that a higher expression level of *ICOS* mRNA significantly predicted response to ICB therapy according to RECIST (Fig. 6B) and a prolonged PFS following initiation of ICB (HR = 0.847 [95%CI 0.725–0.990], *P* = 0.036; Fig. 6C). We then investigated CpG 2 and CpG 4/5 methylation with regard to OS, PFS, and response. However, CpG 2 methylation, as a marker for inflammation that correlated inversely with *ICOS* mRNA expression, was not associated with OS in the TCGA cohort (HR = 2.145 [95%CI 0.227–20.220], *P* = 0.51). Since the Liu et al. cohort does not include DNA methylation data we embraced our previously published UHB ICB case/control patients’ set (*N* = 48, [25]) in order to perform methylation analyses. CpG 2 was neither associated with response (*P* = 0.99) nor PFS (HR = 2.064 [95%CI 0.285–14.941], *P* = 0.47). CpG 4/5 methylation on the other hand, that negatively correlated with *ICOS* mRNA expression by tumor cells and differentiation, was significantly associated with better OS in the TCGA cohort (Cox proportional hazards analysis including CpG 4/5 methylation as continuous variate: HR = 0.227 [95%CI 0.091–0.563], *P* = 0.001; Kaplan–Meier analysis based on an optimized cutoff: *P* < 0.001 (Fig. 6D)). Intriguingly, low CpG 4/5 methylation was significantly associated with responsiveness to ICB (*P* = 0.026, Fig. 6E) and good PFS (Cox proportional hazards analysis including CpG 4/5 methylation as continuous variate HR = 8.095 [95%CI 1.337–49.021], *P* = 0.023; Kaplan–Meier analysis based on an optimized cutoff: *P* < 0.001 (Fig. 6F)) in the UHB ICB case/control study. However, we did not find a correlation between CpG 4/5 methylation and OS in the UHB ICB case/control study that comprised of selected responders and non-responders (HR = 2.309 [95%CI 0.343–15.548], *P* = 0.39). Since our results from the TCGA cohort and the UHB ICB case/control study show that low CpG 4/5 methylation is a biomarker for higher aggressiveness but also better response to ICB, OS differences might be abrogated by these opposing associations. Thus, we expected an association of CpG 4/5 methylation with OS in an ICB cohort that is not comprised of selected responders and non-responders. We developed a qMSP assay that targets CpG 4/5 and tested this hypothesis in an independent cohort of *N* = 123 patients with advanced melanomas treated with ICB (UHB ICB cohort). As expected, in this cohort CpG 4/5 methylation was significantly correlated with OS in a Cox proportional hazards analysis (HR = 1.010 [95%CI 1.000–1.019], *P* = 0.041) and Kaplan–Meier survival analysis based on an optimized cutoff (QMS = 65%; Fig. 6G). On the other hand, we did not find an association of CpG 4/5 methylation with PFS (HR = 0.997 [95%CI 0.988–1.006], *P* = 0.51) or response (*P* = 0.77) in this cohort.

Finally, we tested the independency of *ICOS* CpG 4/5 methylation and *ICOS* mRNA as biomarkers in melanoma. Unfortunately, matched mRNA and methylation data from the same patients’ samples were only available from the TCGA cohort. Therefore, we tested the prognostic value of *ICOS* CpG 4/5 methylation and *ICOS* mRNA with regard to OS in multivariate Cox proportional hazards analysis. We included pathologic stage as an established prognostic biomarker into the model. We found that *ICOS* CpG 4/5 methylation, *ICOS* mRNA, and pathologic stage were independent prognostic biomarkers in melanoma (*ICOS* mRNA HR = 0.995 [95%CI 0.992–0.999], *P* = 0.009; *ICOS* CpG 4/5 methylation: HR = 0.248 [95%CI 0.098–0.623], *P* = 0.003; pathologic stage: HR = 1.424 [95%CI 1.122–1.808], *P* = 0.004). In the UHB ICB case/control study, CpG 4/5 methylation was independently associated with poor PFS when tested together with age, sex, M category, lactate dehydrogenase (LDH), sample type, and anti-PD-1 ICB therapy regimen (HR = 15.488 [2.118–113.263], *P* = 0.007; Supplemental Table S1).

We have previously shown that CpG methylation (CpG site covered by Infinium BeadChip bead cg08460026) of the co-localized *CTLA4* gene predicts response to ICB in melanoma and clear cell renal cell carcinoma [21–23]. Accordingly, we found a significant correlation between CpG 4/5 methylation and *CTLA4* cg08460026 methylation in the TCGA cohort (*ρ* = 0.381, *P* < 0.001, *N* = 470) and in UHB ICB case/control study (*ρ* = 0.391, *P* = 0.006, *N* = 48).

## Discussion

The therapeutic landscape of melanoma is rapidly changing with a plethora of novel immunotherapeutic agents coming to the fore. This will lead to further improvement in the prognosis of melanoma but, however, will also challenge oncologists to dictate the personalized optimal therapy or therapy sequence for the individual patient. In this context, treatment efficacy has to be balanced with treatment safety. Currently, both, anti-PD-1 monotherapy and combination therapy of anti-PD-1 and anti-CTLA-4 are applied in the treatment of melanoma. In a direct comparison of both therapeutic regimens, combination therapy is superior to monotherapy, but at the expense of a higher rate of therapy-mediated adverse events [1]. There is no reliable biomarker that can be incorporated into the treatment decision process of melanoma. Attempts to stratify melanoma therapy by tumoral PD-L1 expression, tumor mutational burden, pre-existing T cell infiltration, and an interferon γ signature are currently under consideration but have not yet been included as companion predictive diagnostics into clinical routine [44]. In previous studies, we identified *CTLA4* methylation as a predictive biomarker for anti-PD-1 and anti-CTLA-4-directed therapy in patients with metastatic melanoma and metastatic clear cell renal cell carcinoma [21–23]. CTLA-4 belongs to the family of CD28 immune checkpoint receptors that includes CD28 and ICOS. In the present study, we comprehensively investigated *ICOS* DNA methylation and mRNA expression with regard to clinicopathological and immune parameters, survival, and response to ICB in melanoma. The rationale of focusing on *ICOS* derived from its CD28/CTLA-4/ICOS family membership, its genomic co-localization with *CTLA4* on chromosome 2 q33.2, and its important dualistic function in balancing pro-tumorigenic and anti-tumorigenic effects thereby representing both, an important immunotherapeutic key target and a promising biomarker candidate for prediction of immunotherapy response.

In the present study, the detailed methylation analysis revealed typical features of a via DNA methylation epigenetically regulated gene, i.e. cell type-specific methylation pattern, inverse correlation with *ICOS* mRNA expression, lower methylation levels in the central promoter region compared to the promoter flanks and intragenic regions, increased expression after treatment with the demethylating agent decitabine, and an association with differentiation. However, we did not find a significant correlation between *ICOS* DNA methylation and ICOS protein expression in melanoma tissue. In accordance to the cell type-specific methylation pattern we found significant correlations with immunologic features, e.g. immune infiltrates and interferon γ. We identified two loci of interests, referred to as CpG 2 and CpG 4/5, whose methylation strongly correlated with immune infiltration (CpG 2) and differentiation and tumor cell-intrinsic *ICOS* mRNA expression (CpG 4/5). While *ICOS* mRNA expression showed a significant association with overall survival, response to PD-1-targeted monotherapy or combined anti-PD-1 and anti-CTLA-4 ICB, and progression-free survival under ICB, we identified *ICOS* hypomethylation as a biomarker of poor prognosis but better response to ICB and prolonged PFS in a case/control study comprised of responders and non-responders and OS in a cohort of ICB treated melanomas. The latter cohort, however, was depleted by responders and non-responders that were already included in the case/control study. This limitation might explain the lack of an association of *ICOS* hypomethylation with response and PFS in this cohort. Taken together, our results suggest that low CpG 4/5 methylation is associated with aggressiveness but higher response to ICB which is in-line with our previous findings regarding methylation of the second member of the CD28 immune receptor family and co-localized gene *CTLA4* in melanoma and clear cell renal cell carcinoma [21–23].

Research has shown, that immune cell infiltrated melanomas exhibit a favorable prognosis and are more susceptible for ICB [45]. Moreover, ICOS has been identified as an indicator of T cell-mediated response to ICB [46]. We found strong positive correlations between *ICOS* mRNA expression and immune infiltrates, which suggest a prognostic and predictive value of *ICOS* mRNA expression. Accordingly, we showed that *ICOS* mRNA expression was associated with improved survival and response to immunotherapy. However, despite the moderate negative correlation between *ICOS* mRNA expression and CpG 2 methylation, we did not detect a prognostic or predictive value of CpG 2 methylation. In the TCGA cohort, the presence of hypermethylation of CpG sites 4/5 was associated with significantly improved overall survival. Considering the inverse correlation with mRNA expression, *ICOS* negative melanomas tend to have an improved prognosis compared to *ICOS* expressing tumors. Of overriding interest, *ICOS* methylation status of combined CpG sites 4/5 demonstrated a predictive value with respect to ICB response in our ICB treated melanoma UHB ICB case/control set. Within this patients’ samples set, hypomethylated tandem CpG sites 4 and 5 were significantly linked to a prolonged PFS. Moreover, methylation status of the tandem CpG sites was predictive for ICB response. This appears at first contradictory considering the data from the TCGA cohort, with hypomethylation being a biomarker of poor prognosis. However, in the survival analysis of the TCGA cohort, we excluded patients who received ICB to prevent a therapy-induced bias on the prognostic value. Following ICB initiation, hypomethylation as an initially unfavorable prognostic marker seems to be contradicted by the positive prediction in terms of ICB therapy response. Hence, the high response to ICB might have overcompensated the negative prognostic value. Of note, this phenomenon of opposite prognostic and predictive value was present with regard to *CTLA4* methylation in melanoma and clear cell renal cell carcinoma [21–23].

While broad evidence exists in terms of ICOS expression on the surface of various immune cell subtypes, little is known regarding tumor-intrinsic ICOS protein expression. Our analyses of RNA-sequencing data from melanoma cell lines and single-cell RNA-sequencing data obtained from melanoma tumor tissue showed that the majority of melanomas do not express *ICOS* mRNA. However, some melanomas exhibit *ICOS* mRNA expression, which correlated with CpG 4/5 methylation. ICOS expression on the protein level by tumor cells was further validated by immunohistochemical staining. Increasing evidence reveals a tumor cell-intrinsic expression of immune receptors, including the CD28 family member CTLA-4 as well as other checkpoint receptors like LAG3, TIGIT, and PD-1 in various malignancies [22, 35, 36, 47, 48]. Of note, while most tumor cell show a cytoplasmic ICOS protein expression, we identified nuclear expression in hepatocytes and in melanoma cells which points towards a hitherto undescribed biological role of ICOS. The traditional concept of transmembrane receptors has already been challenged as nuclear expression has been found for several transmembrane receptors, including the epidermal growth factor receptors EGFR (ERBB1) and HER-2 (ERBB2), fibroblast growth factor receptor 1 (FGFR1), transforming growth factor β receptor 1 (TGFBR1), and insulin like growth factor 1 receptor (IGF1R) (reviewed in: [49–51]). Interestingly, in the early nineties, nuclear EGFR expression has firstly been described in rat hepatocytes (reviewed in: [50, 51]) which is in-line with our finding of nuclear ICOS expression in adjacent hepatocytes from a melanoma liver metastasis. Evidence suggests an involvement of nuclear EGFR expression in gene and cell cycle regulation, phosphorylation of nuclear proteins, and DNA damage repair. Nuclear FGFR1 has further been shown to be involved in neuronal differentiation (reviewed in: [50, 51]). Nuclear expression of the EGFR receptor is associated with poor prognosis across several tumor entities and predictive of poor therapeutic response to targeted therapy (reviewed in: [50, 51]). To what extent such functions can be transferred to ICOS is merely speculative. Hence, this finding is only descriptive and needs to be investigated in further functional studies. If confirmed, targeting ICOS in tumor cells by anti-ICOS mAbs might induce a second mode of action beyond T cell activation.

Despite the strong negative correlation between *ICOS* CpG 4/5 methylation and mRNA expression in melanoma cell lines, we did not find a significant correlation between methylation and ICOS protein expression in melanoma cells. This finding is in accordance with our previously report on *CTLA4* promoter methylation and CTLA-4 protein expression in melanoma [22]. Possible explanations comprise alternative promoter usage, different splice variants, regulation on the level of mRNA (e.g. via RNA modification, long non-coding RNAs [lncRNA], and microRNAs [miRNAs]) as well as differentially regulated protein turnover and translocation. However, the post-transcriptional and post-translational regulation of *ICOS* in tumor cells is beyond the scope of our study and needs further investigation.

Epigenetic markers, such as DNA methylation, are promising biomarkers in modern oncology. Unlike mRNA or protein expression, DNA methylation is stable over time and not subjected to dynamic fluctuations. Further, only small amounts of tissue are required to determine the DNA methylation pattern. Methylation levels are quantifiable and accordingly the assessment is investigator independent.

## Conclusion

In the current study, we demonstrated that ICOS expression is regulated epigenetically via DNA methylation in melanoma. We showed that both, *ICOS* mRNA expression and *ICOS* methylation pattern have prognostic and predictive value with respect to immunotherapy response. Considering the requirements for a biomarker, *ICOS* methylation in particular shows considerable potential. Its predictive potential needs to be investigated in further prospective cohorts. It remains to be evaluated whether a predictive value in relation to ICOS-directed immunotherapy is also conceivable based on *ICOS* DNA methylation status.

## Supplementary Information

**Additional file 1:** **Supplemental Table S1. **UHB ICB case/control study baseline characteristics, associations and correlations with *ICOS *CpG 4/5 methylation, and Cox proportional hazard analysis of progression-free survival.

## Data Availability

The results shown here are in part based on data generated by The Cancer Genome Atlas project (TCGA, http://cancergenome.nih.gov/) and data available under GEO accession numbers GSE103541 or provided by Liu et al. [26] and Tirosh et al. [28]. All other data are available from the corresponding author on reasonable request.

## Abbreviations

6-FAM: 6-Carboxyfluorescein
5-aza-dC: 5-Aza-2-deoxycytidine
95%CI: 95% Confidence interval
APC: Antigen-presenting cell
ATCC: American Type Culture Collection
BHQ-1: Black hole quencher 1
CD278: Cluster of differentiation 278
CpG: Cytosine-phosphate-guanosine
CT: Cycle threshold
CTL: Cytotoxic T lymphocyte
CTLA-4: Cytotoxic T-lymphocyte- associated protein 4
DSMZ: Deutsche Sammlung von Mikroorganismen und Zellkulturen
EDTA: Ethylenediaminetetraacetic acid
FBS: Fetal bovine serum
FFPE: Formalin-fixed and paraffin-embedded
GDC: Genomic Data Commons
GDSC: Genomics of Drug Sensitivity in Cancer
GEO: Gene Expression Omnibus
HEPES: 4-(2-Hydroxyethyl)-1-piperazineethanesulfonic acid
HEX: Hexachlorofluorescein
HR: Hazard ratio
ICB: Immune checkpoint blockade
ICOS: Inducible T cell costimulator
IFNG: Interferon γ
IHC: Immunohistochemistry
LAG-3: Lymphocyte-activation gene 3
LDH: Lactate dehydrogenase
mAb: Monoclonal antibody
MEM: Minimum Essential Medium
MFI: Mean fluorescence intensity
MITF: Microphthalmia transcription factor
NCBI: National Center for Biotechnology Information
NSCLC: Non-small cell lung cancer
OS: Overall survival
PCR: Polymerase chain reaction
PD-1: Programmed cell death protein 1
PFS: Progression-free survival
QMS: Quantitative methylation score
qMSP: Quantitative methylation-specific PCR
qRT-PCR: Quantitative reverse transcription PCR
RECIST: Response Evaluation Criteria in Solid Tumors
RMA: Robust Multi-array Average
RSEM: RNA-Seq by Expectation Maximization
TCGA: The Cancer Genome Atlas
Th: T helper
TIL: Tumor-infiltrating lymphocyte
Treg: Regulatory T cell
UHB: University Hospital Bonn

## References

[1] Carlino MS, Larkin J, Long GV (2021). Immune checkpoint inhibitors in melanoma. Lancet.

[2] Kraehenbuehl L, Weng CH, Eghbali S, Wolchok JD, Merghoub T (2022). Enhancing immunotherapy in cancer by targeting emerging immunomodulatory pathways. Nat Rev Clin Oncol.

[3] Solinas C, Gu-Trantien C, Willard-Gallo K (2020). The rationale behind targeting the ICOS-ICOS ligand costimulatory pathway in cancer immunotherapy. ESMO Open.

[4] Martin-Orozco N, Li Y, Wang Y, Liu S, Hwu P, Liu YJ (2010). Melanoma cells express ICOS ligand to promote the activation and expansion of T-regulatory cells. Cancer Res.

[5] Edwards J, Tasker A, Pires da Silva I, Quek C, Batten M, Ferguson A, et al. Prevalence and Cellular Distribution of Novel Immune Checkpoint Targets Across Longitudinal Specimens in Treatment-naïve Melanoma Patients: Implications for Clinical Trials. Clin Cancer Res. 2019;25(11):3247–58. 10.1158/1078-0432.CCR-18-4011 30777877

[6] Sim GC, Martin-Orozco N, Jin L, Yang Y, Wu S, Washington E (2014). IL-2 therapy promotes suppressive ICOS+ Treg expansion in melanoma patients. J Clin Invest.

[7] Bogunovic D, O’Neill DW, Belitskaya-Levy I, Vacic V, Yu YL, Adams S (2009). Immune profile and mitotic index of metastatic melanoma lesions enhance clinical staging in predicting patient survival. Proc Natl Acad Sci U S A.

[8] Nelson MH, Kundimi S, Bowers JS, Rogers CE, Huff LW, Schwartz KM, et al. The inducible costimulator augments Tc17 cell responses to self and tumor tissue. J Immunol. 2015;194(4):1737–47. 10.4049/jimmunol.1401082 PMC4323681 25576595

[9] Liakou CI, Kamat A, Tang DN, Chen H, Sun J, Troncoso P (2008). CTLA-4 blockade increases IFNgamma-producing CD4+ICOShi cells to shift the ratio of effector to regulatory T cells in cancer patients. Proc Natl Acad Sci U S A.

[10] Vonderheide RH, LoRusso PM, Khalil M, Gartner EM, Khaira D, Soulieres D (2010). Tremelimumab in combination with exemestane in patients with advanced breast cancer and treatment-associated modulation of inducible costimulator expression on patient T cells. Clin Cancer Res.

[11] Yi JS, Ready N, Healy P, Dumbauld C, Osborne R, Berry M (2017). Immune Activation in Early-Stage Non-Small Cell Lung Cancer Patients Receiving Neoadjuvant Chemotherapy Plus Ipilimumab. Clin Cancer Res Off J Am Assoc Cancer Res.

[12] Ng Tang D, Shen Y, Sun J, Wen S, Wolchok JD, Yuan J (2013). Increased frequency of ICOS+ CD4 T cells as a pharmacodynamic biomarker for anti-CTLA-4 therapy. Cancer Immunol Res.

[13] Carthon BC, Wolchok JD, Yuan J, Kamat A, Ng Tang DS, Sun J (2010). Preoperative CTLA-4 blockade: tolerability and immune monitoring in the setting of a presurgical clinical trial. Clin Cancer Res Off J Am Assoc Cancer Res.

[14] Chen H, Fu T, Suh WK, Tsavachidou D, Wen S, Gao J (2014). CD4 T cells require ICOS-mediated PI3K signaling to increase T-Bet expression in the setting of anti-CTLA-4 therapy. Cancer Immunol Res.

[15] Fu T, He Q, Sharma P (2011). The ICOS/ICOSL pathway is required for optimal antitumor responses mediated by anti-CTLA-4 therapy. Cancer Res.

[16] Pauken KE, Sammons MA, Odorizzi PM, Manne S, Godec J, Khan O (2016). Epigenetic stability of exhausted T cells limits durability of reinvigoration by PD-1 blockade. Science.

[17] Ghoneim HE, Fan Y, Moustaki A, Abdelsamed HA, Dash P, Dogra P (2017). De Novo Epigenetic Programs Inhibit PD-1 Blockade-Mediated T Cell Rejuvenation. Cell.

[18] de Vos L, Dietrich J, Strieth S, Bootz F, Dietrich D, Franzen A (2020). PD-1, CTLA4, PD-L1 and PD-L2 DNA methylation in papillary thyroid carcinoma. Immunotherapy.

[19] Hoffmann F, Zarbl R, Niebel D, Sirokay J, Fröhlich A, Posch C (2020). Prognostic and predictive value of PD-L2 DNA methylation and mRNA expression in melanoma. Clin Epigenetics.

[20] Ralser DJ, Klümper N, Gevensleben H, Zarbl R, Kaiser C, Landsberg J, et al. Molecular and Immune Correlates of PDCD1 (PD-1), PD-L1 (CD274), and PD-L2 (PDCD1LG2) DNA Methylation in Triple Negative Breast Cancer. J Immunother. 2021;44(8):319–24. 10.1097/CJI.0000000000000384 34347720

[21] Klümper N, Ralser DJ, Zarbl R, Schlack K, Schrader AJ, Rehlinghaus M (2021). CTLA4 promoter hypomethylation is a negative prognostic biomarker at initial diagnosis but predicts response and favorable outcome to anti-PD-1 based immunotherapy in clear cell renal cell carcinoma. J Immunother Cancer.

[22] Fietz S, Zarbl R, Niebel D, Posch C, Brossart P, Gielen GH (2021). CTLA4 promoter methylation predicts response and progression-free survival in stage IV melanoma treated with anti-CTLA-4 immunotherapy (ipilimumab). Cancer Immunol Immunother CII.

[23] Goltz D, Gevensleben H, Vogt TJ, Dietrich J, Golletz C, Bootz F (2018). CTLA4 methylation predicts response to anti-PD-1 and anti-CTLA-4 immunotherapy in melanoma patients. JCI Insight.

[24] Network CGA (2015). Genomic Classification of Cutaneous Melanoma. Cell.

[25] Fröhlich A, Loick S, Bawden EG, Fietz S, Dietrich J, Diekmann E (2020). Comprehensive analysis of tumor necrosis factor receptor TNFRSF9 (4–1BB) DNA methylation with regard to molecular and clinicopathological features, immune infiltrates, and response prediction to immunotherapy in melanoma. EBioMedicine.

[26] Liu D, Schilling B, Liu D, Sucker A, Livingstone E, Jerby-Arnon L (2019). Integrative molecular and clinical modeling of clinical outcomes to PD1 blockade in patients with metastatic melanoma. Nat Med.

[27] Hannon E, Mansell G, Walker E, Nabais MF, Burrage J, Kepa A (2021). Assessing the co-variability of DNA methylation across peripheral cells and tissues: Implications for the interpretation of findings in epigenetic epidemiology. PLoS Genet.

[28] Tirosh I, Izar B, Prakadan SM, Wadsworth MH, Treacy D, Trombetta JJ (2016). Dissecting the multicellular ecosystem of metastatic melanoma by single-cell RNA-seq. Science.

[29] Schwartz LH, Litière S, de Vries E, Ford R, Gwyther S, Mandrekar S, et al. RECIST 1.1-Update and clarification: From the RECIST committee. Eur J Cancer. 2016;62:132–7. 10.1016/j.ejca.2016.03.081 PMC5737828 27189322

[30] Yang W, Soares J, Greninger P, Edelman EJ, Lightfoot H, Forbes S, et al. Genomics of Drug Sensitivity in Cancer (GDSC): a resource for therapeutic biomarker discovery in cancer cells. Nucleic Acids Res. 2013;41(Database issue):D955–961. 10.1093/nar/gks1111 PMC3531057 23180760

[31] Du P, Zhang X, Huang CC, Jafari N, Kibbe WA, Hou L (2010). Comparison of Beta-value and M-value methods for quantifying methylation levels by microarray analysis. BMC Bioinformatics.

[32] Jung M, Kristiansen G, Dietrich D (2018). DNA Methylation Analysis of Free-Circulating DNA in Body Fluids. Methods Mol Biol Clifton NJ.

[33] Lee JC, Fong L. Agonizing over the stimulatory immune checkpoint ICOS. Clin Cancer Res. 2022;28(17):3633–5. 10.1158/1078-0432.CCR-22-1520 PMC11787823 35792807

[34] Peng C, Huggins MA, Wanhainen KM, Knutson TP, Lu H, Georgiev H (2022). Engagement of the costimulatory molecule ICOS in tissues promotes establishment of CD8+ tissue-resident memory T cells. Immunity.

[35] Mo X, Zhang H, Preston S, Martin K, Zhou B, Vadalia N (2018). Interferon-γ Signaling in Melanocytes and Melanoma Cells Regulates Expression of CTLA-4. Cancer Res.

[36] Niebel D, Fröhlich A, Zarbl R, Fietz S, de Vos L, Vogt TJ (2022). DNA methylation regulates TIGIT expression within the melanoma microenvironment, is prognostic for overall survival, and predicts progression-free survival in patients treated with anti-PD-1 immunotherapy. Clin Epigenetics.

[37] de Vos L, Grünwald I, Bawden EG, Dietrich J, Scheckenbach K, Wiek C (2020). The landscape of CD28, CD80, CD86, CTLA4, and ICOS DNA methylation in head and neck squamous cell carcinomas. Epigenetics.

[38] Sensi M, Catani M, Castellano G, Nicolini G, Alciato F, Tragni G (2011). Human cutaneous melanomas lacking MITF and melanocyte differentiation antigens express a functional Axl receptor kinase. J Invest Dermatol.

[39] Riesenberg S, Groetchen A, Siddaway R, Bald T, Reinhardt J, Smorra D (2015). MITF and c-Jun antagonism interconnects melanoma dedifferentiation with pro-inflammatory cytokine responsiveness and myeloid cell recruitment. Nat Commun.

[40] Ballotti R, Cheli Y, Bertolotto C (2020). The complex relationship between MITF and the immune system: a Melanoma ImmunoTherapy (response) Factor?. Mol Cancer.

[41] Vivas-García Y, Falletta P, Liebing J, Louphrasitthiphol P, Feng Y, Chauhan J (2020). Lineage-Restricted Regulation of SCD and Fatty Acid Saturation by MITF Controls Melanoma Phenotypic Plasticity. Mol Cell.

[42] Landsberg J, Kohlmeyer J, Renn M, Bald T, Rogava M, Cron M (2012). Melanomas resist T-cell therapy through inflammation-induced reversible dedifferentiation. Nature.

[43] Reinhardt J, Landsberg J, Schmid-Burgk JL, Ramis BB, Bald T, Glodde N (2017). MAPK Signaling and Inflammation Link Melanoma Phenotype Switching to Induction of CD73 during Immunotherapy. Cancer Res.

[44] Nishino M, Ramaiya NH, Hatabu H, Hodi FS (2017). Monitoring immune-checkpoint blockade: response evaluation and biomarker development. Nat Rev Clin Oncol.

[45] Petitprez F, Meylan M, de Reyniès A, Sautès-Fridman C, Fridman WH (2020). The Tumor Microenvironment in the Response to Immune Checkpoint Blockade Therapies. Front Immunol.

[46] Xiao Z, Mayer AT, Nobashi TW, Gambhir SS (2020). ICOS Is an Indicator of T-cell-Mediated Response to Cancer Immunotherapy. Cancer Res.

[47] Wang X, Yang X, Zhang C, Wang Y, Cheng T, Duan L (2020). Tumor cell-intrinsic PD-1 receptor is a tumor suppressor and mediates resistance to PD-1 blockade therapy. Proc Natl Acad Sci U S A.

[48] Klümper N, Ralser DJ, Bawden EG, Landsberg J, Zarbl R, Kristiansen G, et al. LAG3 (LAG-3, CD223) DNA methylation correlates with LAG3 expression by tumor and immune cells, immune cell infiltration, and overall survival in clear cell renal cell carcinoma. J Immunother Cancer. 2020;8(1):e000552. 10.1136/jitc-2020-000552 PMC7174079 32234847

[49] Chen PC, Kuo YC, Chuong CM, Huang YH (2020). Niche Modulation of IGF-1R Signaling: Its Role in Stem Cell Pluripotency, Cancer Reprogramming, and Therapeutic Applications. Front Cell Dev Biol.

[50] Lo HW (2010). Nuclear mode of the EGFR signaling network: biology, prognostic value, and therapeutic implications. Discov Med.

[51] Brand TM, Iida M, Li C, Wheeler DL (2011). The nuclear epidermal growth factor receptor signaling network and its role in cancer. Discov Med.

