# Supplementary material for: ICOS DNA methylation regulates melanoma cell-intrinsic ICOS expression, is associated with melanoma differentiation, prognosis, and predicts response to immune checkpoint blockade
Source: Biomark Res. 2023 Jun 1;11:56. doi: 10.1186/s40364-023-00508-2 (PMC10233860; doi:10.1186/s40364-023-00508-2)
Supplement: Supplementary file 1 — Additional file 1: Supplemental Table S1. UHB ICB case/control study baseline characteristics, associations and correlations with ICOS CpG 4/5 methylation, and Cox proportional hazard analysis of progression-free survival. [file 40364_2023_508_MOESM1_ESM.pdf]

Supplemental Table S1: UHB ICB case/control study baseline characteristics, associations and correlations with *ICOS* CpG 4/5 methylation (continuous variate), and Cox proportional hazard analysis (uni- and multivariate) of progression-free survival.

				Univariate Cox proportional hazards		Multivariate Cox proportional hazards	
**Characteristics**	**Patients**	***ICOS* CpG 4/5 methylation [95% CI]**	***P* value**[†]	**Hazard ratio [95% CI]**	***P* value**	**Hazard ratio [95% CI]**	***P* value**
**Age (mean, range)**	66 (29-89)	47.3% [40.3%-54.3%]	*P* = 0.35	0.996 [0.971-1.022]	*P* = 0.76	1.000 [0.967-1.034]	*P* = 0.99
**Sex**			*P* = 0.82		*P* = 0.67		*P* = 0.39
Female	15 (31%)	49.2% [34.7%-63.7%]		Reference group		Reference group	
Male	33 (69%)	46.5% [38.3%-54.8%]		1.208 [0.504-2.896]		1.636 [0.539-4.964]	
**M category**			*P* = 0.61		*P* = 0.96		*P* = 0.84
M1a	6 (13%)	43.1% [13.6%-72.7%]		Reference group		Reference group	
M1b	8 (17%)	38.1% [20.2%-56.1%]		1.384 [0.305-6.284]		2.112 [0.294-15.164]	
M1c	26 (54%)	50.0% [40.9%-59.0%]		1.161 [0.285-4.728]		1.944 [0.302-12.530]	
M1d	8 (17%)	51.0% [25.7%-76.4%]		1.319 [0.432-4.031]		1.143 [0.353-3.702]	
**LDH**			*P* = 0.96		*P* = 0.20		*P* = 0.095
Normal	29 (60%)	47.5% [38.6%-56.3%]		Reference group		Reference group	
Elevated	19 (40%)	47.1% [34.6%-59.6%]		1.680 [0.757-3.730]		2.432 [0.857-6.898]	
**Sample type**			*P* = 0.26		*P* = 0.42		*P* = 0.81
Distant metastasis	10 (21%)	51.3% [33.1%-69.5%]		Reference group		Reference group	
Primary or locally recurrent tumor	7 (15%)	52.7% [37.5%-67.8%]		0.465 [0.090-2.403]		0.706 [0.105-4.752]	
Cutaneous metastasis	17 (35%)	38.4% [26.7%-50.0%]		0.815]0.266-2.497]		0.825 [0.221-3.083]	
Lymph node metastasis	14 (29%)	52.7% [37.1%-68.2%]		1.450 [0.493-4.265]		1.257 [0.366-4.324]	
**Therapy**			*P* = 0.60		*P* = 0.026		*P* = 0.016
anti-PD-1	20 (42%)	46.0% [34.8%-57.2%]		Reference group		Reference group	
anti-PD-1 and anti-CTLA-4 (combination or sequential)	28 (58%)	48.2% [38.7%-57.7%]		0.350 [0.139-0.881]		0.226 [0.067-0.758]	
***ICOS* CpG 4/5 methylation**	48 (100%)	47.3% [40.3%-54.3%]	NA	8.095 [1.337-49.021]	*P* = 0.023	15.488 [2.118-113.263]	*P* = 0.007

[†] Mann-Whitney *U* test (sex, LDH, therapy), Kruskal-Wallis test (M category, sample type), Spearman's ρ correlation (age); NA: not applicable
